# Clinical usefulness of measuring endotoxin activity on ICU admission

**DOI:** 10.1186/cc10641

**Published:** 2012-03-20

**Authors:** A Murai, H Ishikura, T Nishida, Y Irie, T Kamitani, R Yuge, T Kitamura, T Umemura

**Affiliations:** 1 Fukuoka University Hospital, Fukuoka City, Japan

## Introduction

According to the Surviving Sepsis Campaign, diagnosis of sepsis and infection is urgent, therefore rapid diagnostic tools play a major role in the management of septic patients. The endotoxin activity (EA) assay (EAA) is one of those tools based on the ability of antigen-antibody complexes to prime neutrophils for an augmented respiratory burst response [1]. EAA has been used widely in patients who had suspected infection in the emergency room and ICU, but the clinical usefulness of measuring EAA in the diagnosis of sepsis in critically ill patients is not yet clear.

## Methods

We performed an observational cohort study in critically ill patients in the ICU of a tertiary care hospital. We investigated the correlation between EA levels and blood concentration of endotoxin measured by the chromogenic limulus amoebocyte lysate (LAL) assay, causative microorganism identified in laboratory culture, procalcitonin (PCT), soluble CD14 subtype (named presepsin), IL-6, antithrombin, protein C, thrombomodulin, lactate, disseminated intravascular coagulation scores in both the Japanese Ministry of Health and Welfare and the Japanese Association for Acute Medicine, and severity of illness at ICU admission.

## Results

We enrolled 49 subjects. There was no significant correlation between EA levels and endotoxin concentration measured by LAL assay. There were no significant difference in the EA levels of the Gram-negative infection patients and the others. The diagnostic value of EA levels was investigated using ROC curve analysis. For the diagnosis of sepsis, area under the curve of EA levels, PCT, presepsin, IL-6 and CRP were calculated as 0.76, 0.83, 0.89, 0.88 and 0.72, respectively. Both the EA levels and ICU mortalities of the patients who met the criteria for severe sepsis were significantly higher than those of the patients who did not have sepsis (0.44 ± 0.21 vs. 0.22 ± 0.17, *P *= 0.0004; EA levels, 33% vs. 5%, *P *= 0.022; ICU mortalities). There was a positive relationship between EA levels and thrombomodulin (*r *= 0.30, *P *= 0.049), EA levels and lactate (*r *= 0.31, *P *= 0.028), and EA levels and SOFA score (*r *= 0.34, *P *= 0.02). There was a negative relationship between EA levels and platelet counts (*r *= -0.34, *P *= 0.018), EA levels and antithrombin (*r *= - 0.41, *P *= 0.004), and EA levels and protein C (*r *= -0.38, *P *= 0.010).

## Conclusion

EA levels in the patients on ICU admission correlated with disease severity. Moreover, we strongly suggested that EAA may have the potential to assess organ dysfunction with sepsis, especially coagulopathy.
